# Medical Waste-Sorting and Management Practices in Five Hospitals in Ghana

**DOI:** 10.1155/2020/2934296

**Published:** 2020-03-04

**Authors:** Robert Ohene Adu, Samuel Fosu Gyasi, David Kofi Essumang, Kenneth Bentum Otabil

**Affiliations:** ^1^Department of Civil and Environmental Engineering, University of Energy and Natural Resources, Sunyani, B.A., Ghana; ^2^Department of Basic and Applied Biology, University of Energy and Natural Resources, Sunyani, B.A., Ghana; ^3^Department of Chemistry, School of Physical Sciences, College of Agriculture and Natural Sciences, University of Cape Coast, Cape Coast, Ghana

## Abstract

Hospital waste management in Ghana faces the risk of cross-contamination from the lack of thorough sorting of the waste at the points of generation, codisposal of hazardous and nonhazardous waste types, and use of open-fire pits and substandard incinerators for burning infectious waste. This has increased the potential for the spread of infections and chemical pollutants. A cross-sectional study was conducted in five hospitals in Ghana to assess behavioral patterns on waste sorting and the effectiveness of hospital waste management in Ghana. A total of 250 questionnaires were distributed purposively to some staff of the five hospitals to assess workers' perceptions on medical waste sorting and handling. Additionally, focused group discussions and transect walks were adopted to examine the current collection, storage, treatment, and disposal methods used in the health facilities. Chi-square analyses showed significant differences in waste-sorting behavior based only on occupation (*p* < 0.0001, *n*=180) and not on gender, education, or experience in the health sector. Even though contaminated sharps were separated into brown safety boxes, color coding for other infectious waste containers was inconsistent across the health facilities. The study revealed that incineration is still the modal method of treatment in Ghanaian hospitals and therefore new approaches such as an engineering approach were required to minimize its environmental effects. It is recommended that periodic in-service training workshops be held for healthcare staff on the right source-segregation of medical waste, in order to facilitate the effective and safe handling, transport, treatment, and disposal of waste from health facilities.

## 1. Introduction

Healthcare waste is increasing in quantity and in diversity worldwide [1, 2]. Modern healthcare facilities have become large establishments, producing not only hazardous wastes but also general waste such as stationery wastes and food wastes. The World Health Organization (WHO) defines healthcare waste to include all the wastes generated within healthcare facilities, research centers, and laboratories related to medical procedures, as well as from minor and scattered sources, including wastes produced in the course of healthcare undertaken in the home such as home dialysis, self-administration of insulin, and recuperative care [1].

Indeed, the World Health Organization [3] estimates that around 85% of healthcare wastes are nonhazardous (comparable to domestic waste), while 10% are infectious (cultures and stocks of infectious agents, wastes from infected patients, wastes contaminated with blood and its derivatives, discarded diagnostic samples, infected animals from laboratories, and contaminated materials and equipment) and anatomic wastes (recognizable body parts and carcasses of animals) and the remaining 5% is hazardous healthcare waste (chemical, radioactive). Whereas an ageing population is the major driver of the increased medical waste generation in the advanced world, in the developing world, changing diagnostic trends especially toward the increased use of disposable equipment in medical care, as well as improved living standards and access to healthcare, have contributed to the increased generation of healthcare waste [4].

However, improper management of hospital waste has brought untold occupational, environmental, and public health hazards to hospital workers, patients, and communities worldwide [5]. A number of needle-stick injuries have been reported among hospital workers and scavenger families while handling infected waste mixed with other types of waste [6]. A study by [7] revealed that 7,550 needle-stick and sharps injuries were reported among 8,645 health workers in Taiwan, 66.7% of these injuries involving a contaminated hollow-bore needle. In Sub-Saharan Africa, the reuse of contaminated syringes and needles in medical care has accounted for 5% of HIV infections [8]. In 2001, more than 300 million tons of injection-related wastes were generated during a mass immunization of 6 million children against measles across 6 West African countries [9]. Such waste generation levels could create a particular health hazard to health workers and the public. Children playing around waste dump sites have contracted diseases such as Hepatitis and HIV through percutaneous injuries from contact with infected sharps waste. Unsterilized syringes were estimated by the WHO to cause 80,000 to 160,000 cases of HIV, 2.3 to 4.7 million cases of hepatitis C, and 8 to 16 million cases of hepatitis B every year [10, 11]. Although there have been marked improvements since then, unsafe injections still accounted for between 16,939 and 33,877 new HIV infections, for between 157,592 and 315,120 hepatitis C infections, and nearly 1.7 million hepatitis B infections as of 2010 [12]. A study by Blenkharn and Odd [13] has shown that sharp waste items such as hypodermic needles have sometimes found their way inside containers meant for soft clinical wastes and this has caused injuries in hospital environments.

In Ghana, whereas 0.5 kg of municipal solid waste (MSW) is generated per person per day, an average of 1.5 kg/bed/day of healthcare waste is generated by health facilities in Ghana [14, 15]. This nearly compares with healthcare facilities in developing cities like Dhaka in Bangladesh that generate about 1.9 kg/bed/day [16]; about 2.7 kg/bed/day in Tehran, Iran [17]; and between 0.84 and 5.8 kg/bed/day in Dar-es-Salaam, Tanzania [18]. A number of field visits to various health facilities in Ghana were undertaken by a multisectoral working group established by Ghana's EPA, together with Ghana's Ministry of Local Government and Rural Development. This cross-sectoral working group came up with a number of guidelines for managing healthcare and veterinary waste in Ghana [15]. As part of the guidelines, hospital wastes were classified into five broad categories from A to E with their appropriate color codes (see [Supplementary-material supplementary-material-1] in Supplementary Materials).

The segregation of hospital waste at the points of generation has been studied to markedly reduce the spread of infectious agents [4]. Thus, the effective management of medical waste must begin at source, that is, at the wards and units, and must continue through the secondary stage at the hospital premises. .

### 1.1. Research Problem

Most health facilities in Ghana do not sort their waste. In the capital region of Ghana for instance, 83% of health facilities do not sort their waste [19]. Where they do, the infectious fraction sorted will still end up at the landfill sites, mixed with the general municipal waste if the health facility has no incinerator [19] or open-fire pit. Considering the fact that when infectious waste is mixed with general waste, it must all be considered infectious [4, 15, 20, 21], enormous volumes of solid waste will have to be specially treated as hazardous in Ghana and that will be hugely expensive.

Currently, some health facilities in Ghana benefit from the communal collection system spearheaded by a private company, Zoomlion Ghana Limited, on behalf of municipal assemblies [22]. The company collects and mixes the nonhazardous wastes with hazardous wastes from many healthcare facilities and sends them to central disposal facilities. Since no pretreatment may occur at the disposal sites, the combined waste can still remain infectious.

Most incinerators in use in Ghana are not equipped with Air Pollution Control (APC) systems [23]. The cost involved in procuring and running APC equipment discourages its use. As a result, lots of noxious organic and inorganic pollutants are released in the flue gas.

The determination of what constitutes infectious waste differs from jurisdiction to jurisdiction. The unnecessary classification of solid wastes in many hospitals as infectious has affected choice and cost of waste management [24] and led to huge volumes of medical waste requiring special treatment such as incineration.

Efficient sorting of medical waste at source is critical to any effective waste management strategy in any jurisdiction around the world. Thus, even the most advanced systems of medical waste management still require some sorting of the waste at the points of generation. Therefore, obtaining information about waste-sorting and management practices by healthcare workers is extremely important.

This study therefore was aimed at assessing medical waste-sorting and management practices in 5 major hospitals in Ghana.

## 2. Methodology

### 2.1. Study Areas

**Figure 1:**
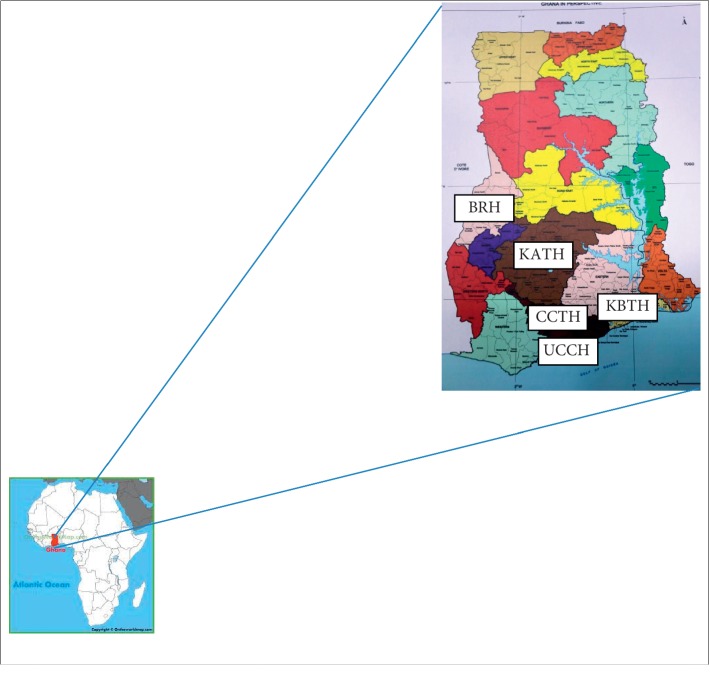
Map of Ghana showing selected hospitals (Google images).

The study was conducted in 5 major hospitals in Ghana, West Africa. These included Korle Bu Teaching Hospital in Accra (designated KBTH); the Komfo Anokye Teaching Hospital in Kumasi (designated KATH); the Cape Coast Teaching Hospital (designated CCTH); the Brong Regional Hospital in Sunyani (designated BRH); and the University of Cape Coast hospital in Cape Coast (designated UCCH) as shown in Figure 1 below.

### 2.2. Study Design

A cross-sectional study approach was employed and the study was conducted from June 2018 to April 2019.

### 2.3. Study Population and Sample

A sample size of two hundred and fifty (250) hospital workers was purposively chosen from a study population of 11,220 health workers in 5 hospitals in Ghana, within a 10% margin of error, following the sampling formula of *n*_*o*_=(*Z*^2^*pq*/*d*^2^), and *n*=(*n*_*o*_/(1+(*n*_*o*_/*N*))), adopted by Cochran [25]. Individuals recruited for this study included key management and other staff members from the above-mentioned health institutions who had worked in various jobs for various years.

### 2.4. Study Approach

A mixed-method research approach was adopted involving the qualitative and the quantitative stages. This was to eliminate any bias inherent in any method or data source [26]. The first stage was the qualitative stage which included transect walks. These entailed systematic walks along defined paths around waste receptacles within and outside the hospitals' premises, as well as around on-site waste disposal and treatment facilities of the 5 different health institutions at different times. The purpose was to explore sanitation conditions by observing, interacting with workers, listening, watching, asking questions, and taking photographs.

The quantitative phase involved the administration of a structured questionnaire and this assisted in generating information on behavioral patterns on waste sorting from a population of hospital staff across different departments of five major hospitals in Ghana who were exposed to the risks of infectious wastes. With the help of daily visits, a representative sample of staff, purposively sampled across the different departments of the 5 selected hospitals, were recruited and interviewed using structured and semistructured questionnaires ([Supplementary-material supplementary-material-1] in Supplementary Materials). Within the cohort of professions selected, workers were randomly sampled for the questionnaire administration. Questions asked included their social demographic background, their perceptions on hospital waste management, and the current performance of their hospital incinerators and other treatment facilities among many others. The 250 questionnaires were administered in all the 5 hospitals selected. Respondents included nurses, pharmacists, diagnostic staff, biostatisticians, and technical as well as other staff. Focused group discussions were held with key informants in the waste management sectors of the hospitals ([Supplementary-material supplementary-material-1] in Supplementary Materials).

### 2.5. Theoretical Framework

**Figure 2:**
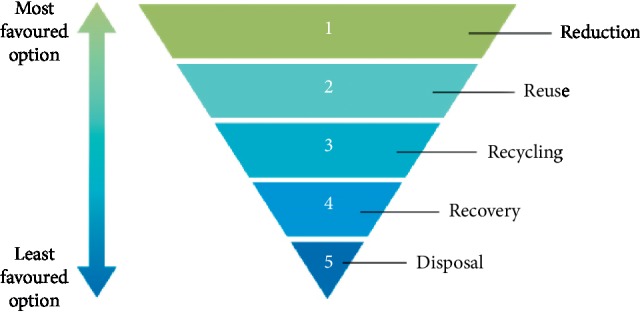
Hierarchy of waste management [27].

This study adopted, as its theoretical framework, the hierarchy of waste management (shown in Figure 2) as contained in the recommendations of Agenda 21 of the 1992 World Conference on Environment and Development organized by the UN in Rio De Janeiro, Brazil [28]. The Agenda 21 recommendations stipulate, among others, the following:

- The prevention and minimization of waste production
- The reuse or recycling of waste to the extent possible
- The treatment of wastes by safe and environmentally sound methods
- The disposal of final residues by landfill in confined and carefully designated sites.

Further, Agenda 21 stresses that waste producers should be responsible for the treatment and final disposal of their wastes; where possible each community should dispose of its wastes within its own boundaries. The safe management of wastes produced is formulated within the framework of a national plan for healthcare waste management.

### 2.6. Ethical Issues

Ethical approvals were obtained from the Scientific and Technical Committee and the Institutional Review Board of the Korle Bu Teaching Hospital in Accra, Ghana ((KBTH-STC/IRB/000110/2018); the Ethical Review Committee of Cape Coast Teaching Hospital (Ref: CCTHERC/EC/2018/35); and the Committee on Human Research, Publication and Ethics of the School of Medical Sciences/Komfo Anokye Teaching Hospital in Kumasi (Ref: CHRPE/AP/604/18). Participants' confidentiality was ensured as their names were not included in the questionnaires. A consent form was designed with simple and clear language for easy reading. All procedures were performed in accordance with the declaration of Helsinki on human subject protection.

### 2.7. Data Analyses

Responses from the structured questionnaires were manually validated, coded, and entered in Microsoft Excel (2010). Categorical variables were analyzed using chi-square at a significance level, *p* < 0.05 (95% confidence interval), with the help of GraphPad Prism, version 5 (GraphPad Software Inc). This was to establish whether there were any significant differences in waste-sorting behavior among the selected health workers based on gender, occupation, educational level, or experience in the health sector.

## 3. Results

General information on the 5 hospitals indicated that the teaching hospitals, KBTH, KATH, and CCTH, had the highest staff strengths, patient attendances, and bed complements (Table 1).

### 3.1. Transect Walks

**Figure 3:**
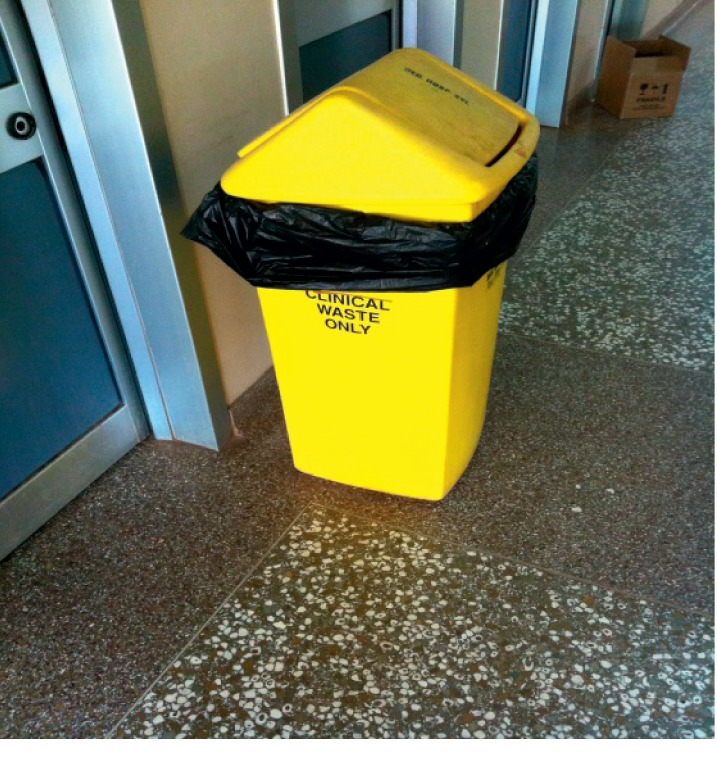
“Clinical waste only” container and sharps box at the Brong Regional Hospital.

During transect walks at the 5 major hospitals, certain observations on their waste disposal practices were made. At the Brong Regional Hospital, yellow containers with black plastic linings for only clinical waste and brown safety boxes for sharps waste were placed right outside the wards and units (Figure 3).

**Figure 4:**
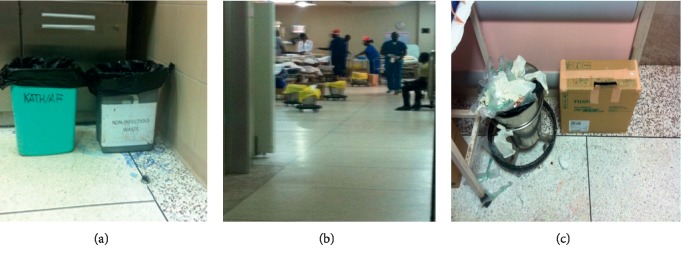
Waste sorting at various units of KATH. (a) Hematology unit. (b) Resuscitation unit. (c) Radiography unit.

At the Komfo Anokye Teaching Hospital, most of the wards and units had two containers, one for soft infectious waste and a brown safety box for sharps waste, that is, needles, syringes, and other sharp items (Figure 4).

**Figure 5:**
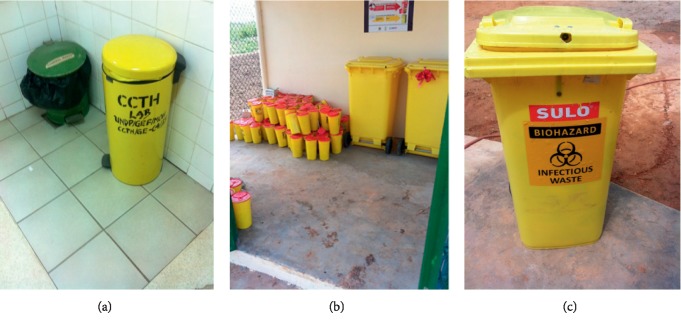
Waste sorting at CCTH. (a) Sorted waste at lab. (b) Sorted waste for central autoclave. (c) Infectious waste.

At the Cape Coast Teaching Hospital (CCTH), waste sorting was practiced at the various wards and units as well as in the laboratories (Figure 5).

**Figure 6:**
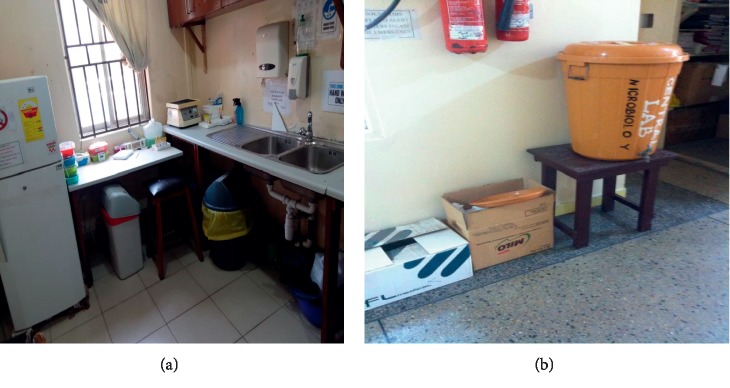
Waste sorting at KBTH. (a) Immunology Lab. (b) Central Lab Corridor.

At the Korle Bu Teaching Hospital, waste was sorted inside the labs and wards into infectious with red liners and noninfectious with yellow or black liners (Figure 6).

### 3.2. Collection, Storage, and Disposal of Hospital Waste

The general wastes generated in the surveyed hospitals were collected and stored in 240 L bins that stood outside the wards and offices but within the hospitals' premises. These were emptied once or twice a day by waste collectors and sent to bigger containers which were emptied by third-party companies.

**Figure 7:**
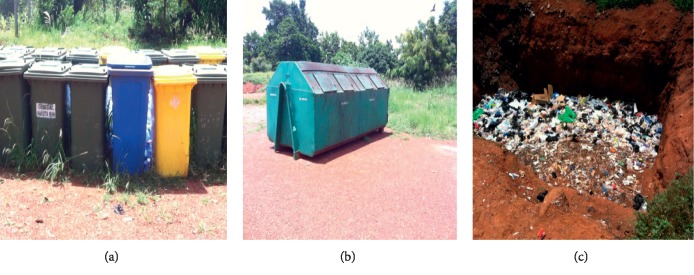
B.A Regional Hospital. (a) 240 L containers. (b) 12 m^3^ container. (c) An open pit for infectious waste.

The Brong Regional Hospital (BRH) had secondary 240 L bins and a bigger container with a capacity of 12 m^3^ which gets filled with general waste every 2 days and is emptied by the Sunyani Municipal Assembly (Figure 7). There is an open pit for burning infectious waste as the incinerator was nonfunctional at the time of this study (Figure 7).

**Figure 8:**
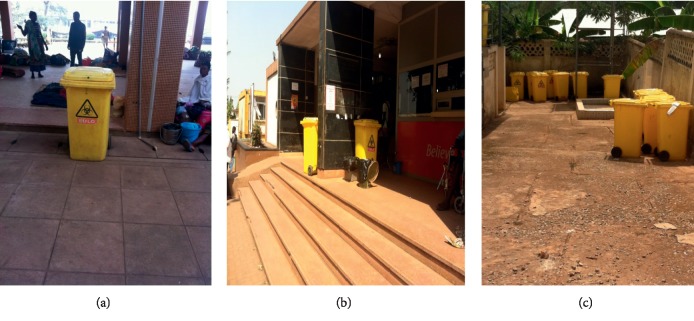
Komfo Anokye Teaching Hospital. (a, b) Waste storage bins outside wards. (c) Storage bins by incinerator.

Secondary 240 L containers at Komfo Anokye Teaching Hospital (KATH) were placed in the vicinity of the hospital and were emptied once or twice a day (Figure 8).

**Figure 9:**
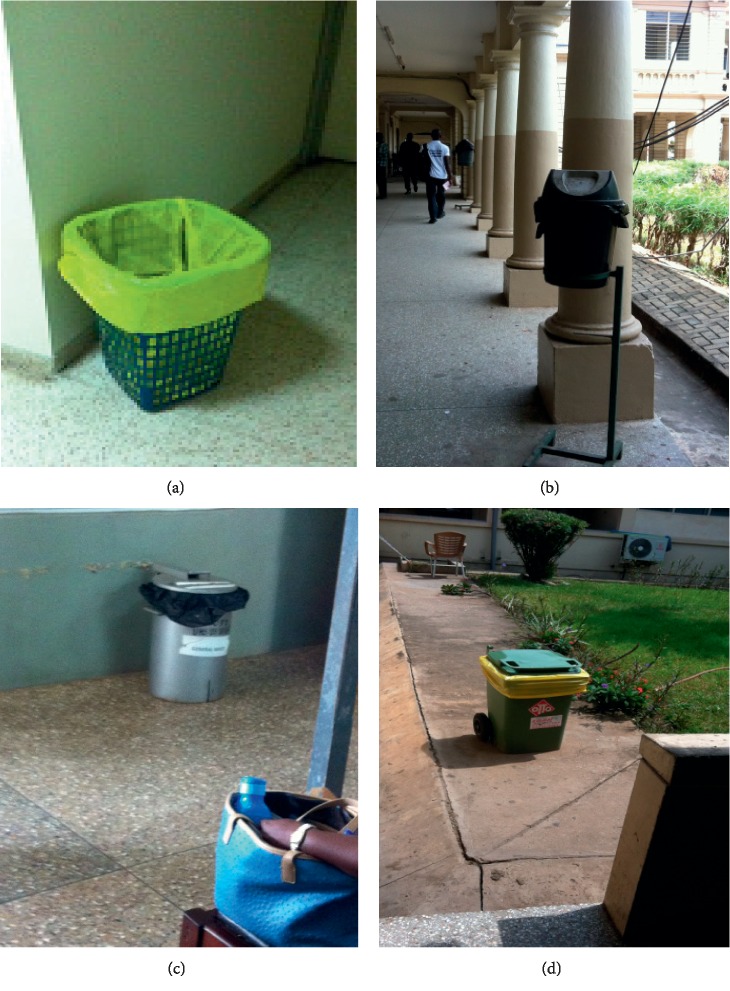
General waste bins at Korle Bu Teaching Hospital. (a) Yellow-lined basket outside an office. (b) Black-lined bin at corridor. (c) Black-lined bin outside the Child Care Department. (d) Yellow-lined bin by corridor.

At the Korle Bu Teaching Hospital, waste containers were placed outside the wards with different color coding. Yellow- and black-lined bins were placed outside the wards and offices for general waste (Figure 9).

### 3.3. Waste Treatment Practices

The study revealed that 4 out of the 5 hospitals surveyed had an incinerator installed for burning infectious waste. These were the KBTH, KATH, BRH, and UCCH. The only exception was the Cape Coast Teaching Hospital (CCTU) which had a central autoclave installed for infectious waste sterilization at 134°C (Table 2).

**Figure 10:**
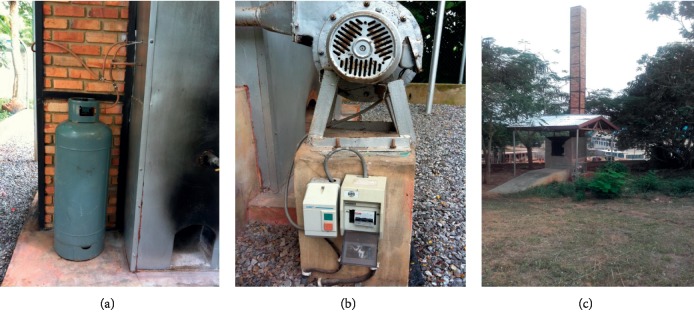
Locally made incinerator at UCC Hospital, Cape Coast. (a) 52-kg LP gas (auxiliary fuel) cylinder. (b) Blower. (c) Chimney.

For some of the hospitals surveyed, the waste incinerators installed were either broken down or operating under substandard conditions. Most of the incinerators surveyed had chimneys without air pollution control devices (APCDs) mounted. At the University of Cape Coast hospital, the incinerator, though locally made and nonengineered, still functioned reliably, burning infectious waste into ash (Figure 10).

The ash produced was added to the general waste containers for disposal. The incinerator uses liquefied petroleum gas (LPG) as auxiliary fuel with a fuel efficiency of about 10cycles/cylinder. The cycle time for complete combustion was about 5 hours. The chimney had no APCD equipment.

**Figure 11:**
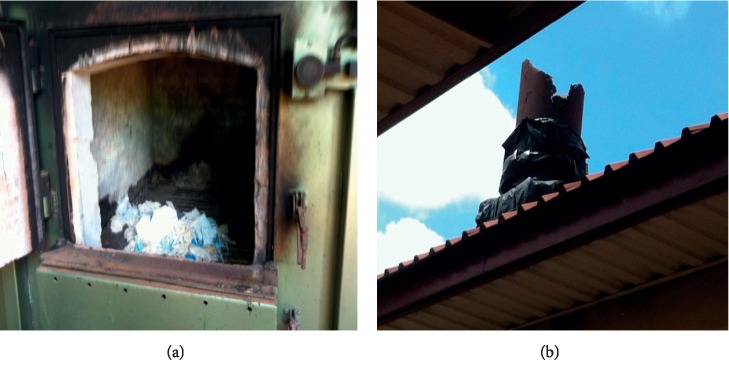
Imported incinerator at Brong Regional Hospital, Sunyani. (a) Combustion chamber. (b) Chimney without APCD.

At the Brong Regional Hospital (BRH), the incinerator was out of service and had a rusted chimney which had no air pollution control device (APCD) (Figure 11).

**Figure 12:**
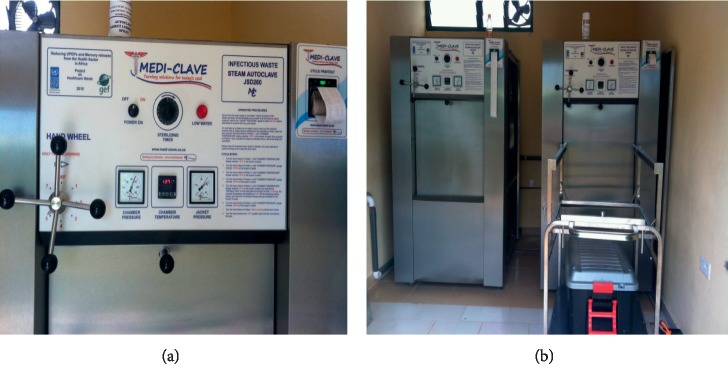
Central autoclaves for infectious waste sterilization at the CCTH, Cape Coast, Ghana.

At the Cape Coast Teaching Hospital (CCTH), waste incineration was phased out, and a steam-based treatment method, known as autoclaving, was now used to treat only the infectious blood-borne components of the waste including cotton pads, soiled bandages, and blood-stained needles and syringes. Two central autoclaves, known as “Mediclaves,” were installed under the UNDP Medical Waste project currently being piloted among 5 health facilities in Ghana (Figure 12).

This waste treatment process is a predisposal technique and uses steam to sterilize the waste at 134°C. As a result, no ash is produced. The sterilized wet waste is then added to the general waste produced at the hospital for onward disposal. Other types of waste such as expired drugs and amputated body parts are not treated here but sent to a nearby facility at Nkamfoa, a suburb of Cape Coast, for burning or burial.

**Figure 13:**
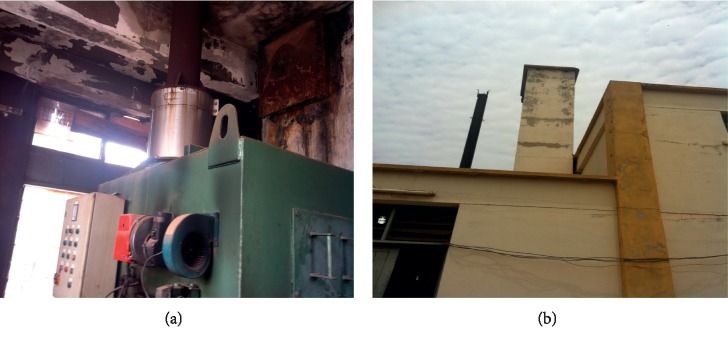
Anatomic waste incinerator at KATH. (a) Combustion chamber. (b) Chimney.

**Figure 14:**
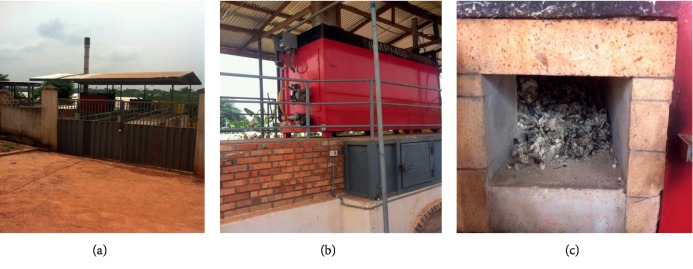
An LPG-powered Addfield MP400 incinerator for infectious waste sterilization at KATH. (a) Chimney. (b) Combustion Chamber. (c) Ash after combustion.

At the Komfo Anokye Teaching Hospital, two incinerators were installed, one for burning anatomic wastes (“Incinco” type) installed since 1996 and the other for burning infectious waste (Addfield MP 400, Britain) recently installed (Figures 13 and 14).

This MP400 incinerator runs on LP gas and has 3 compartments: a primary burner (C) which operates at a temperature of 600°C; a secondary burner (A) which operates at 1200°C, and a hot hearth (H) which enhances the bricks. It has 3 thermocouples which give temperature signals in the three chambers (Figure 14). About 30–50 kg of infectious waste is incinerated per cycle. There is no air pollution control device fitted.

**Figure 15:**
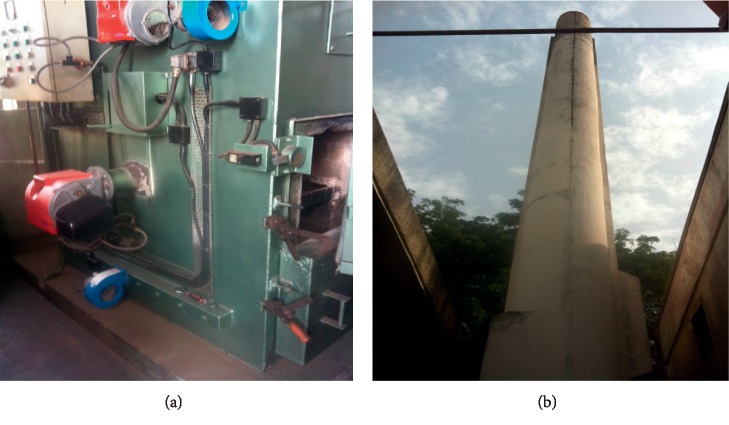
Incinerator for burning infectious waste at KBTH. (a) Combustion chamber. (b) Chimney.

At the Korle Bu Teaching Hospital, there was a mechanized incinerator named “Incinco” installed for burning infectious waste (Figure 15). This two-chambered incinerator had been operating for 27 years as of March 2019 and has a 20-foot-tall chimney stack for releasing exhaust gases. The cycle time for burning is 5-6 hours every day for all 7 days in the week and it runs on diesel fuel.

Results of focused group discussions on medical waste incineration included statements such as the following:

 
*KATH Incinerator*. “This incinerator is mainly for burning anatomic wastes such as placentas and amputations. Because of the sensitive nature of such wastes, it is not ethical to have pictures taken of charred bodies and placentas inside the combustion chamber.”
 
*UCCH Incinerator*. “Because this incinerator is situated in the vicinity of the hospital, as well as close to primary and junior high schools, we usually burn the waste early in the morning, so that the smoke does not become a nuisance during school and working hours.”

### 3.4. Solid Waste Generation Estimates from Selected Hospitals

General information on the 5 health facilities was obtained from the biostatistics or records units and included staff strength, bed complement, and out-patient department (OPD) attendances (Table 3). As data on total waste generation rates at the hospitals were unavailable, the daily rates were computed using the bed complements of the selected hospitals and an average value of 1.5 kg/bed/day, adopted from findings by a collaborative survey by the EPA-Ghana and the Ministry of Local Government and Rural Development of Ghana [15].

### 3.5. Analyses of Waste-Sorting Behavior

Out of the 250 questionnaires administered, a total of 202 questionnaires were returned by the respondents. Twenty-seven (27) were received from UCCH, thirty-four (34) from CCTH, forty-nine (49) from BRH, forty-four (44) from KATH, and forty-eight (48) from KBTH.

The analyses of waste-sorting behavior of staff based on their gender, occupation, educational qualification, and working experience in the health sector showed that there were no statistically significant differences except for occupation. The results of the cross-tabulation showing variations in the responses of the respondents are presented in [Supplementary-material supplementary-material-1] in Supplementary Materials.

**Figure 16:**
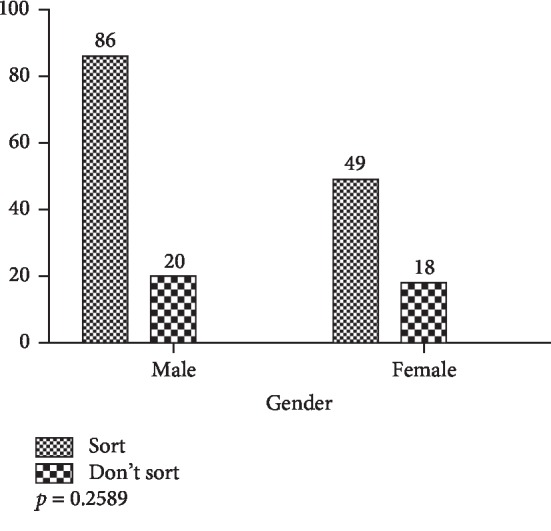
Waste-sorting behavior based on gender.

Based on the gender of respondents, results demonstrated that of the 173 valid responses received from staff, of the 5 hospitals, there were no statistically significant differences in waste-sorting behavior (*p*=0.2589, *X*^2^ test). There were more male respondents (106) than females (67) and more males sorted their waste compared to females (86 and 49, resp.) (Figure 16).

**Figure 17:**
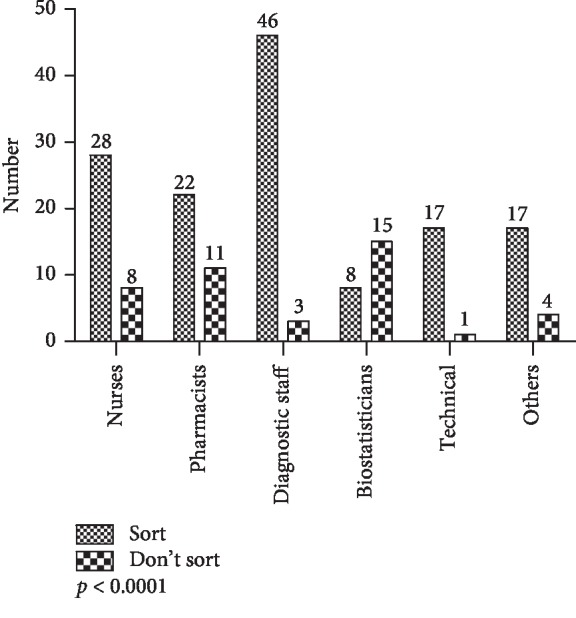
Waste-sorting behavior by occupation.

On the basis of occupation, of the 180 valid responses from 6 groups (nurses, pharmacists, diagnostic staff, biostatisticians, technical staff, and other workers), there were statistically significant differences in waste-sorting behavior of the groups (*p* < 0.0001, *X*^2^ test). The results are presented in Figure 17.

**Figure 18:**
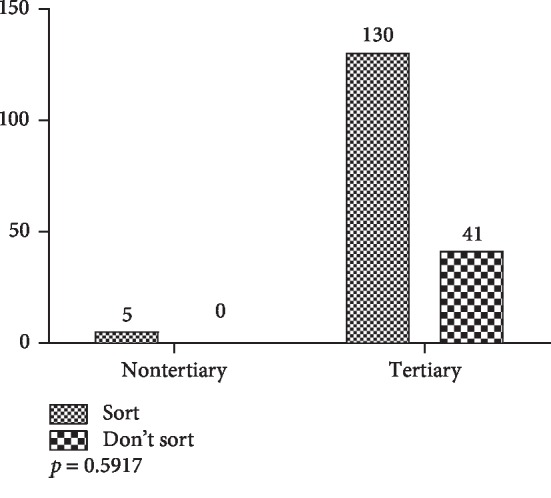
Waste-sorting behavior by qualification.

On educational qualification, the study demonstrated that of the 176 respondents, there were no statistically significant differences in waste-sorting behavior of respondents with tertiary and nontertiary qualifications (*p*=0.2112, *X*^2^ test). The results are presented in Figure 18.

**Figure 19:**
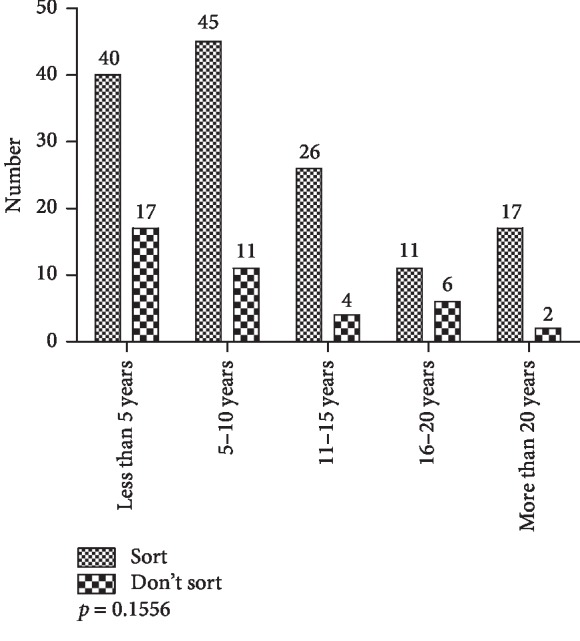
Waste-sorting behavior by working experience.

The analysis of the responses based on the working experience of respondents in the study showed that there were no statistically significant differences (*p*=0.1556, *X*^2^ test) in their waste-sorting behavior (Figure 19).

## 4. Discussion

During transect walks to the 5 hospitals in this study, it was observed that even though medical waste sorting at the points of generation was fairly practiced, safe management was still challenged by the lack of consistent color coding for separate waste types. Studies by the US EPA have recommended that the safe handling of infectious hospital waste should begin at the points of generation, to wit, at the wards and units, where the potential for cross-contamination and disease infection is greatest [4]. Therefore, it is imperative that sorting of hospital waste begins at the wards and units, using separate receptacles and different color codes for the infectious waste fractions, the sharps waste, and the noninfectious waste types and that adequate safeguards and labels for the high-risk waste are provided to prevent patients and hospital staff from contacting them. A study by [29] of two hospitals in Tanzania showed that at least 25% of medical waste in the two hospitals was not sorted at source. Thus, there was still a potential for disease infection at the wards and units and also at the storage points when sorted by waste handlers, as well as at disposal points by scavengers.

Among the 4 criteria used in the analyses of waste-sorting behavior (i.e., gender, occupation, qualification, and working experience), significant differences in waste-sorting behavior were apparent only on the basis of occupation (*p* < 0.0001). Among diagnostic staff (medical lab scientists, radiographers, etc.) and nurses working in high-risk areas, there seemed to be a higher consciousness of the need to separate infectious waste from noninfectious waste, compared to other occupations like biostatistics staff. This is fairly consistent with a study by [30] on attitudes of health workers toward biomedical waste management in a hospital in Oman in which statistically significant differences in attitude were established between sampled laboratory technicians, doctors, nurses, and housekeeping staff toward stricter rules in implementation of biomedical waste management. The finding on working experience is also implied in another study by [31] on health workers in primary health centers in India in which their working experiences were fairly evenly distributed and their knowledge and awareness were lacking on the biomedical waste management.

This study demonstrated that the existing incinerators in the five hospitals investigated were overstretched and operated above their capacity. The situation is made worse because of the lack of proper sorting, as these incinerators were not designed for the types and quantities of feedstock they receive. The findings in this study are consistent with that of [32] who demonstrated that of the few healthcare facilities that had incinerators the equipment was simply inoperable due to overusage. When waste is properly sorted, only the infectious and pathological waste fractions (categories B2, B3, and C of hospital waste, See [Supplementary-material supplementary-material-1] in Supplementary Materials) in yellow containers, also known as clinical waste and representing about 10% of all hospital waste, will be incinerated. This will reduce the load on the incinerators in use and therefore extend their service life. Therefore, the proper education and training of staff and proper indicators are necessary to help in the proper sorting.

Several organizations including the World Health Organization and Ghana's Environmental Protection Agency as well as Health Care Without Harm (HCWH) recommend incineration technology as a temporary method for the treatment of hospital waste [19]. However, a study by [33] revealed that even though incineration of waste produced double the emissions compared to nonincineration treatment methods, its products required 30 times less space for landfilling than products of nonincineration technologies. Besides, it had a lower carbon footprint than nonincineration technologies [33]. Incineration, therefore, cannot be left out of an integrated waste management strategy for medical waste, especially for a developing country like Ghana where landfilling is increasingly challenged by a demand for other land uses. The pathogenic nature of medical waste and the huge advantage of volume reduction of the waste offered by incineration make engineered incineration a more benign choice over other treatment options. The effective incineration of hospital waste certainly leaves behind bottom ash which is reduced greatly in volume and which is more sterile in nature and can safely be handled together with municipal solid waste. Fly ash when trapped in filters mounted in incinerator chimneys can be incorporated in road base materials for road construction and similar uses. Preliminary information from literature suggested that the existing incinerators in most Ghanaian hospitals were often loaded and operated beyond the required percentage of infectious waste meant for incineration [19]. As a result, some incinerators were out of service. This was corroborated by primary data, collected from the selected hospitals in Ghana and by transect walks to selected incineration facilities.

Another observation from this study is that all the hospital waste incinerators were run intermittently. This is similar to observations by [34] in a study in Taiwan that compared dioxin and furan emissions between 4 medical waste incinerators and 10 municipal waste incinerators. The frequent start-up and shut down operations of medical waste incinerators could lower incineration temperatures and cause an increased formation of persistent organic pollutants (POPs) such as dioxin-like PCBs, dioxins, furans, and PAHS, which are carcinogenic [34]. This necessitates the installation of air pollution control devices (APCDs) on incinerators; however, none of the incinerators observed in the present study had any APCDs.

A Ministry of Health [35] study on healthcare waste management in Ghana proposed, as a long-term measure, the limiting of the number of incinerators in Ghana to a few, high-capacity, ones that could be shared among districts/regions. These should be fitted with APCDs that meet international standards. This could assure regular operation and monitoring. Hospitals in Ghana that are equipped with incinerators are from Level C or District/Municipal hospitals and higher up [35]. A number of larger hospitals, namely regional, teaching, and specialist hospitals, are equipped with either imported or local brick incinerators [35]. Besides these incinerators, attempts have been made in research to design prototypes of incinerators with improvements in the burning procedure of hospital waste and in the postcombustion ash management [35].

Whereas the hospital waste incineration in the advanced world is currently being phased out following the Stockholm Convention of 2001, it is still the preferred waste treatment method in hospitals in the developing world [5], and Ghana is no exception. Concerns in developed countries over releases from incinerator exhausts of persistent organic pollutants (POPs)—chiefly dioxins and furans (PCDDs/Fs), dioxin-like PCBs (polychlorinated biphenyls), and polycyclic aromatic hydrocarbons (PAHs)—and over heavy metal releases like mercury (Hg) and lead (Pb) have discouraged incineration in favor of nonincineration technologies such as autoclaving, steam disinfection, chemical disinfection, alkaline hydrolyses, and microwaving [5]. In developing countries, however, challenges with the effective segregation of hospital waste, with high costs of these advanced steam systems and the potential risk of disease infection from the reuse of contaminated sharps [8, 36] make incineration still the preferred option of medical waste management, particularly for the hazardous component. Recent advances in exhaust gas cleaning systems should now allay any fears over incinerator use, if these are fitted onto existing incinerators. The prospect of pathogen destruction makes incineration a preferred treatment option for hospital waste, especially for the infectious waste types which are nonrecyclable.

## 5. Conclusions

A cross-sectional study of waste-sorting and management practices in five hospitals in Ghana has shown that even though there were attempts to segregate hospital waste, particularly in the high-risk areas, the lack of a uniform color coding and labeling system for the different categories of hospital waste affects the efficiency of collection and handling and the integrity of the final waste treatment processes. Significant differences in waste-sorting behavior among health staff were apparent only on the basis of occupation or work area. A number of incinerators for burning infectious waste are either not functioning or are operated outside their capacities or appropriate uses. Current incinerators are unable to inactivate pathogens. Chemical agents like PCDDs/Fs and PCBs are likely released in the exhausts, which calls for the need to install air pollution control devices (APCDs). It is recommended that refresher training courses are periodically organized for health workers to conscientize them on laboratory and general health safety. There is a need for an integrated approach to healthcare waste management in Ghana that will entail the coordinated efforts of the assemblies as well as the Ministry of Health and private companies contracted to collect, transport, and dispose of waste. Since 80–85% of wastes generated in health facilities are of no risk and comparable to domestic waste, they can be separated and handled together with the municipal waste streams while the infectious ones are treated specially, either on-site or sent to designated engineered incinerators.

## Figures and Tables

**Table 1:** General information about 5 hospitals.

Facility	Staff strength	Bed complement	Patient attendance/day
KBTH	5,000+	1,449	1,042
KATH	3,567	1,023	1,347
CCTH	1,327	400	420
BRH	1,012	330	270
UCCH	314	75	281

**Table 2:** Waste treatment facilities at the 5 hospitals.

Facility	Type of incinerator/facility	Auxiliary fuel type	Waste incinerated kg/cycle	Air pollution control	Incineration cycle time
KBTH	Incinco	Diesel	10–50	In-built filter	5-6 hours
KATH	Incinco	Diesel	5–10	In-built filter	2–5 hours
	MP 400	LPG	30–50	No	½–1 hour
CCTH	Autoclave	Steam	—	—	—
BRH	Incinco	Diesel	—	No	—
UCCH	Locally made	LP gas	—	No	5 hours

**Table 3:** General information and solid waste generation estimates from 5 hospitals.

Facility	Staff strength	Bed complement	Patient attendance/day	Estimated solid waste generation (kg/day)
KBTH	5000+	1,449	1,042	2,174
KATH	3,567	1,023	1,347	1,535
CCTH	1,327	400	420	600
BRH	1012	330	270	495
UCCH	314	75	281	113
Total	**11,220+**	**3,277**	**3,360**	**4,917**

## Data Availability

The authors declare that data for this work will be available upon request.

## Supplementary Materials

Supplementary Materials Appendix 1: questionnaire 1. Appendix 2: questionnaire for focused group discussions. Appendix 3: Classification and color coding of hazardous and nonhazardous healthcare waste in Ghana. Appendix 4: Cross-tabulation data for the 4 chi-square analyses.
