# Supplementary material for: Medical Waste-Sorting and Management Practices in Five Hospitals in Ghana
Source: J Environ Public Health. 2020 Mar 4;2020:2934296. doi: 10.1155/2020/2934296 (PMC7073495; doi:10.1155/2020/2934296)
Supplement: Supplementary Materials — Appendix 1: questionnaire 1. Appendix 2: questionnaire for focused group discussions. Appendix 3: Classification and color coding of hazardous and nonhazardous healthcare waste in Ghana. Appendix 4: Cross-tabulation data for the 4 chi-square analyses. [file 2934296.f1.pdf]

Appendix I: Questionnaire 1

Behavioral Patterns on Medical Waste Management in Five Hospitals in Ghana

Name of Health Facility……………………………………………………………………

Municipality ………………………………… Region ……………………………………

Section A: Respondents' Socio-demographic data

1. What is your position? [a] Administrator [b] Clinician [c] Nurse [d] Waste Control Manager [e] Waste Collector [f] Pharmacist [g] Incinerator Operator [h] Engineer [i] Environmental Health Officer [j] other – specify
2. How old are you? [a] Less than 20yrs [b] 21-30 [c] 31-40 [d] 41-50 [e] 51-60 [e] 60+
3. What is your gender [a] Male [b] Female
4. What is your educational level [a] Primary [b] Secondary [c] Tertiary [d] Standard 7 [e] No education
5. How long have you worked in this hospital [a] Less than 5 yrs [b] 5-10 yrs [c] 11-15 yrs [d] 16-20 yrs [e] 20+ yrs
6. How long have you worked in the health sector [a] Less than 5 yrs [b] 5-10 yrs [c] 11-15 yrs [d] 16-20 yrs [e] 20+ yrs

Section B: Information on Health Facility

7. What is the prescribing level of your facility? [a] Teaching/Specialist [b] Regional [c] District/Municipal [d] Private Hospital [e] Other –specify ……………….
8. What is the bed capacity of your health facility? [a] 0-120 [b] 121-250 [c] 251–300 [d] 300 +
9. What is the OPD attendance per day? [a] 0-120 [b] 121-250 [c] 251–300 [d] other-specify
10. What is total staff strength of your hospital [a] 1-100 [b] 101-200 [c] 201–300 [d] 301 – 400 [e] 400+

Section C: Information on Health Facility's Waste Management

11. How much hospital waste is generated daily in your facility? [a] 0-50 kg [b]51-100 kg [c] 101-150 kg [d] 150 + kg
12. What fraction of the waste is infectious? [a] 0-10% [b] 11-15% [c]16-20 % [d] 21-25% [e] 25+%
13. What is the sharps' content of your waste? [a] 0-10% [b] 11-15% [c]16-20 % [d] 21-25% [e] 25+%
14. Do you segregate your waste at the points of generation? [a] Yes [b] No
15. Do you have a separate container for infectious waste at the wards/units? [a] Yes [b] No
16. Do you have a secondary container on site for infectious waste? [a] Yes [b] No
17. If yes, how often is the secondary container emptied? [a] twice a day [b] once a day [c] once in 2 days [d] once in 3 days [e] other (specify)………..
18. Do you make any contact with waste bins at the wards/units/transfer points? [a] Yes [b] No
19. If yes, how? [a] Direct dropping of waste [b] Emptying of waste [c] moving of waste bin [d] other-specify
20. What do you protect yourself with? [a] Gloves [b] Protective coat [c] Boots [d] None [b] Other-specify
21. Do you have an incinerator? [a] Yes [b] No
22. Do you have an Autoclave sterilizer? [a] Yes [b] No
23. If yes, which items are sent to the autoclave? [a] Infectious waste [b] surgical instruments [c] all the waste generated [d] none [e] expired drugs [f] other- specify
24. Do you have an open-fire pit for burning hospital waste? [a] Yes [b] No

25. If yes, which of the waste types is sent to the open-fire pit? [a] Infectious [b] expired drugs [c] all the waste generated [d] none [e] other- specify

Appendix II – Questionnaire for Focused Group Discussions

Behavioral Patterns on Medical Waste Management in Five Hospitals in Ghana

Name of Health Facility……………………………………………………………………

Municipality ………………………………… Region ……………………………………

Incinerator Design and Operation

1. What type of incinerator is in use?
2. Which of these items is/are present in your incinerated waste? :
    i) Pressurized gas containers? [ ] Yes [ ] No
    ii) Reactive chemical waste? [ ] Yes [ ] No
    iii) Silver salts? [ ] Yes [ ] No
    iv) Photographic wastes? [ ] Yes [ ] No
    v) Radiographic wastes? [ ] Yes [ ] No
    vi) Halogenated plastics (PVC, etc)? [ ] Yes [ ] No
    vii) Broken thermometers? [ ] Yes [ ] No
    viii) Used batteries? [ ] Yes [ ] No
    ix) Lead-lined wooden panels? [ ] Yes [ ] No
    x) Sealed ampoules? [ ] Yes [ ] No
3. What is the plastic content of the incinerated waste?
4. Where is it fabricated?
5. How long has the incinerator been in service?
6. Can you describe its current state?
7. How many combustion chambers does the incinerator have?
8. What fuel type is used for ignition?
9. What is the waste load per cycle of incineration?
10. How many cycles per week of incineration is carried out?
11. What is the incineration cycle time?
12. How often is maintenance work done?
13. Of what material is the incinerator wall?
14. How many layers has the incinerator wall
15. What is the total thickness of incinerator wall?
16. What is the chimney height of the incinerator?
17. What is the inner cross-sectional area of the chimney?
18. How is the combustion temperature measured?
19. What is the primary combustion temperature?
20. What is the secondary combustion temperature?
21. Is the incinerator pre-dried before the waste is loaded?
22. At what temperature is infectious waste loaded?
23. How much auxiliary fuel is consumed per cycle?
24. What is the rough budget estimate of the entire set-up?

25. Is the flue gas treated after combustion?
26. What type of Air Pollution Control (APC) device is fitted?
27. How is the incinerator bottom ash disposed of?

Appendix III

Classification and color coding of Non-Hazardous Health Care Waste in Ghana

Type	Classification	Description/examples	Color Coding
A	General Waste	Similar to domestic waste(food waste, sweepings etc)	Black
B	Infectious waste	In-/out-patient or animal waste containing pathogenic microbes (blood, lab waste, cotton swabs etc)	Yellow
	B.1 Sharps waste	Sharp-edged wastes contaminated with blood or body fluids from injection rooms, surgical units etc (surgical blades, needles, scalpels, syringes, ampoules, test tubes, pipettes, glass instruments)	Puncture-resistant containers and yellow plastic bags
	B.2 Patient /Animal waste	Wastes (other than sharps) generated from in-/out-patient activities which may be stained with blood or body fluids from surgical operations, injection room etc (soiled cotton wool, used dressings/bandages, gloves, gauze, linen, infusion sets, blood transfusion bags, urine, faeces, lab waste).	Yellow plastic bags and containers
	B.3 Culture/Specimen	Clinical specimen, human tissue and laboratory culture (e.g. experimental specimen (animals), tissue culture, urine, stool, faeces from laboratory) .	Yellow plastic bags and containers
C	Pathological / Organic Human /Animal Tissue	Amputations and other body tissues from surgical operations, autopsy (post-mortem), birth requiring special treatment for ethical and aesthetic reasons [Internal body organs, amputated limbs, placentas/ fetus, human liquid wastes (e.g. urine, blood products)].	Yellow plastic bags and containers
D	Hazardous waste	Similar to industrial hazardous waste, requiring special treatment (pharmaceutical, laboratory, organic substances, heavy metals and other chemical contamination)	Brown plastic bags and containers
	D.1 Pharmaceutical waste	Wastes from the pharmacy units [Expired drugs (solid/liquid, plastic or glass containers, residuals of drugs in chemotherapy that may be cytotoxic, genotoxic, mutagenic or carcinogenic].	Brown plastic bags and containers
	D.2 Photographic Chemical waste	Waste material (solid or liquid) produced from image processing at the radiology department (X-ray photographic film, Photographic developer, fixer solution)	Brown plastic containers (To be recycled/reused, or neutralised)
	D.3 Radioactive waste	Any solid, liquid, or pathological waste contaminated with radioactive isotopes of any kind (Spent radiation sources, radium needles, technetium generators etc).	Brown containers with radioactive symbol
	D.4 Laboratory waste	Spent chemicals from research and analytical laboratories, and pharmaceutical companies (Acid, Alkali, organic substances, Solvents, and heavy metals).	Brown containers with appropriate labels

	D.4.1 Acids	Hydrochloric acid, glacial acetic acid, chromosulphuric acid, oxalic acid	Brown container with Acid label
	D.4.2 Alkalis	Sodium hydroxide, Potassium hydroxide	Brown container with Alkali label
	D.4.3 Solvents	Methanol, Ethanol, Chloroform, Xylene, and Acetone	Brown container with Solvent label
	D.4.4 Organic substances	Hexamine, paraffin, Phenol, resorcin and Polyvinyl chloride tape.	Brown container with Organic label
	D.4.5 Heavy Metals	Mercury, Lead, cadmium, etc	Brown container with Heavy Metal label
E	Incinerator ash and sludge	Waste generated from the combustion of hospital waste which will have to be disposed of in a landfill site (Incinerator fly and bottom ash and its residues Leachate) .	Yellow metal containers labelled "Ash" or "Ash"

(Adapted from MLGRD, 2002)

Appendix IV: Cross-tabulation Data for the 4 Chi-Square Analyses

1. Waste-Sorting Behavior by Gender (1 degree of freedom)

Gender		Number who sort	Number who do not sort	Total
Male	Observed	86	20	**106**
	Expected	82.7	23.3	
Female	Observed	49	18	**67**
	Expected	52.3	14.7	
**Total**		**135**	**38**	**173**

2. Waste-Sorting Behavior by Profession (5 degrees of freedom)

Occupation/Profession		**Number who sort**	**Number who do not sort**	**Total**
Nurses	Observed	28	8	36
	Expected	27.6	8.4	
Pharmacists	Observed	22	11	33
	Expected	25.3	7.7	
Diagnostic Staff	Observed	46	3	49
	Expected	37.6	11.4	
Biostatisticians	Observed	8	15	23
	Expected	17.6	5.4	
Technical	Observed	17	1	18
	Expected	13.8	4.2	
Others	Observed	17	4	21
	Expected	16.1	4.9	
**Total**		**138**	**42**	**180**

3. Waste-Sorting Behavior by Qualification

**Qualification**		**Number who sort**	**Number who do not sort**	**Total**
Non-tertiary	Observed	5	0	**5**
	Expected	3.84	1.16	
Tertiary	Observed	130	41	**171**
	Expected	131.16	39.84	
**Total**		**135**	**41**	**176**

4. Waste-Sorting Behavior by Working Experience in the Health Sector

**Working Experience**		**Number who sort**	**Number who do not sort**	**Total**
Less than 5 years	Observed	40	17	57
	Expected	44.26	12.74	
5 – 10 years	Observed	45	11	56
	Expected	43.49	12.51	
11 – 15 years	Observed	26	4	30
	Expected	23.30	6.70	
16-20 years	Observed	11	6	17
	Expected	13.20	3.80	
20 + years	Observed	17	2	19
	Expected	14.75	4.25	
**Total**		**139**	**40**	**179**
